# An Uncommon Coexistence of Dural and Intraventricular Meningiomas

**DOI:** 10.7759/cureus.54510

**Published:** 2024-02-20

**Authors:** Afwaan Faizal, Sakthi Ganesh Subramonian, Aashika Parveen Amir, Dinesh Babu Jawahar

**Affiliations:** 1 Radio-Diagnosis, Saveetha Medical College and Hospitals, Saveetha Institute of Medical and Technical Sciences (SIMATS) Saveetha University, Chennai, IND

**Keywords:** headache, magnetic resonance imaging, neurofibromatoses, alanine, meningioma

## Abstract

Meningiomas, originating from the meninges encasing the brain and spinal cord, are the most prevalent primary intracranial tumors, constituting around 40% of all such tumors. These tumors primarily manifest within the dura mater, the outermost meningeal layer, and occasionally in locations such as the ventricular system. However, the concurrent presence of dural and intraventricular meningiomas is exceedingly rare. It could be challenging to tell them apart from metastases. We present a case of a middle-aged female with chronic headaches, where magnetic resonance imaging (MRI) revealed two distinct supratentorial lesions, one dural and the other intraventricular. Surgical excision was successfully performed, and histopathological analysis confirmed the presence of meningiomas in both locations, and subsequent referral was made for comprehensive management, encompassing radiotherapy and chemotherapy. This case underscores the significance of advanced imaging modalities, particularly MRI, in diagnosing and assessing intricate brain tumors.

## Introduction

Among primary brain and other central nervous system (CNS) tumors, meningiomas account for 39.7% of cases, making them the most prevalent primary CNS tumor [1]. According to the WHO CNS5, meningioma is classified as a single category, with 15 subgroups reflecting its wide range of morphological characteristics [2]. These tumors typically appear in the dura mater, the top layer of the meninges, although they can also occur in other places including the ventricular system.

While intraventricular meningiomas account for a very small portion of instances, dural meningiomas are more frequent [3]. As a result, the presence of both these tumors forming in a single patient creates an uncommon clinical situation that is rife with diagnostic and therapeutic challenges. Here, we report the case of a female patient in her 50s presenting with chronic headaches, diagnosed with coexisting dural and intraventricular meningiomas. Detailed radiological and histopathological analyses confirmed the diagnosis. Surgical resection remains the mainstay of treatment, with adjuvant therapies considered for atypical cases.

## Case presentation

**Figure 1:**
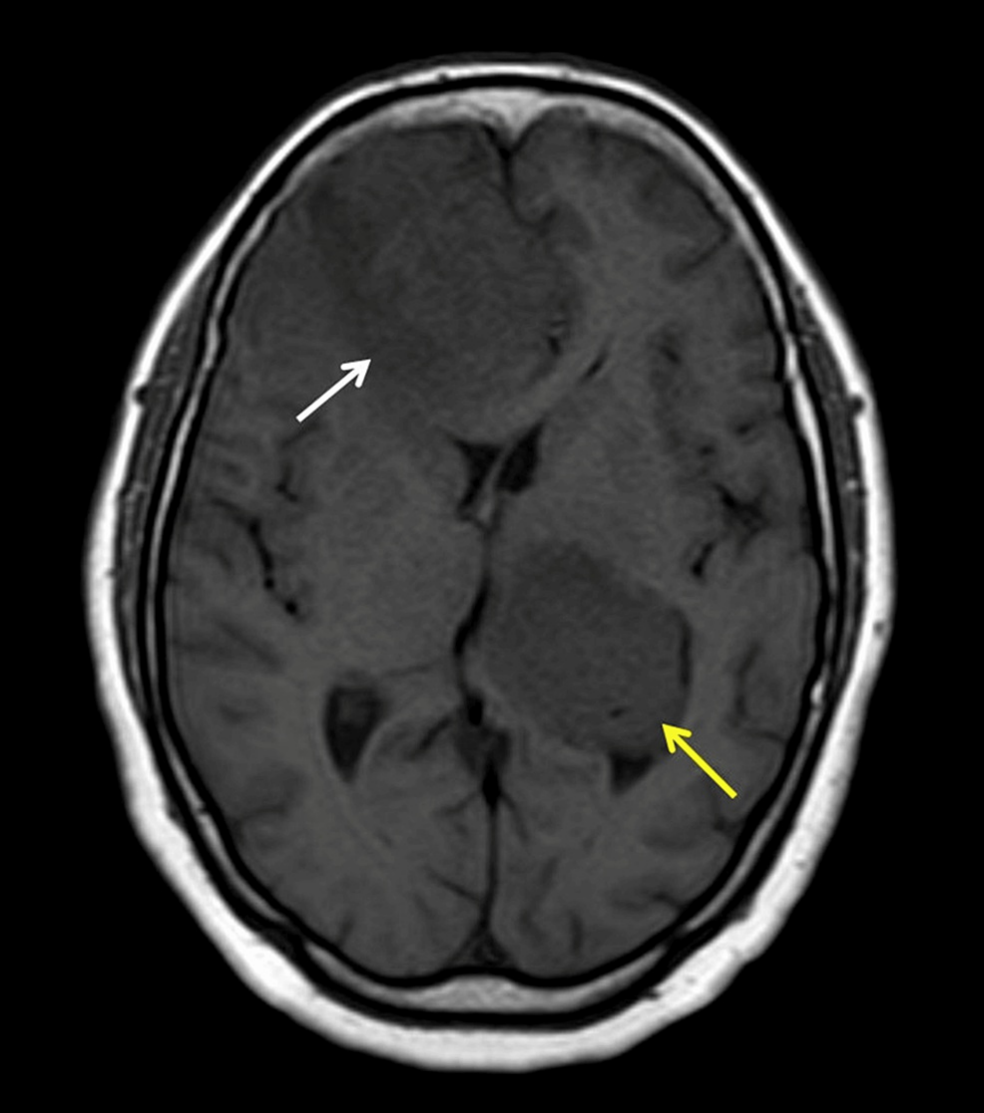
Axial T1 weighted MRI shows coexisting dural meningioma (white arrow) and intraventricular meningioma (yellow arrow)

A female patient in her 50s presented with a history of chronic headaches lasting over 11 months, which had progressively worsened in intensity. The headaches were described as constant, dull, and aching in nature and predominantly localized to the frontal and temporal regions of the head. The patient reported no specific triggers for the headaches, which persisted throughout the day and often interfered with daily activities. Over-the-counter pain medications provided minimal relief, and the patient noted no associated symptoms, such as nausea, vomiting, photophobia, or phonophobia. The patient did not have a history of seizures or any other sensory or motor symptoms, such as paresis, paralysis, hypertonia, or clonus. The patient denied any recent changes in vision, hearing, or cognitive function. Visual acuity was within normal limits. She had no notable family history, particularly concerning meningiomas and neurofibromatosis type 2. Anosmia, seizures, vertigo, or behavioral abnormalities were also not present. On clinical examination, she had no neurocutaneous markers. Standard blood and urine investigations were unremarkable. The decision to pursue brain imaging was made to eliminate intracranial pathology as a potential cause of the headaches. MRI brain was done, which revealed two supratentorial lesions, a dural lesion broad-based toward the falx cerebri was seen along the right frontal convexity, measuring approximately 44x42x40 mm, and an intraventricular lesion was seen epicentered in the atrium of the left lateral ventricle, measuring 39x40x40 mm (Figure 1).

**Figure 2:**
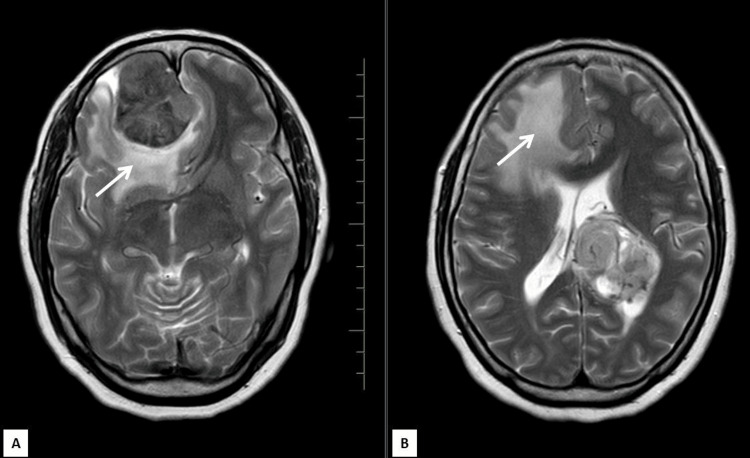
Axial T2 weighted MRIs (A,B) show internal heterogeneous signal changes and signs of mass effect in both lesions with perilesional edema seen in the dural-based lesion (white arrow)

**Figure 3:**
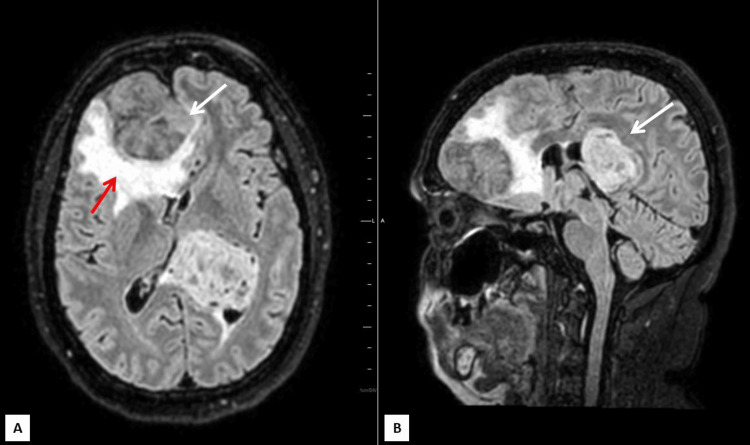
FLAIR magnetic resonance axial (A) and sagittal (B) images show internal heterogeneous signal changes and signs of mass effect in both lesions (white arrows) with perilesional edema seen in the dural-based lesion (red arrow) FLAIR: fluid-attenuated inversion recovery

**Figure 4:**
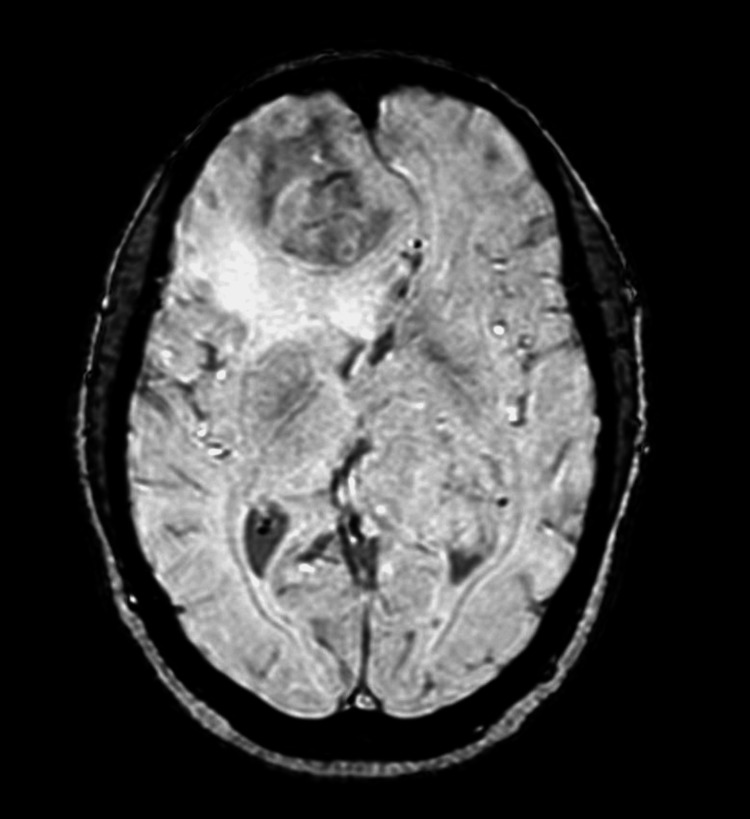
Axial SWI image shows no obvious evidence of internal blooming artifacts SWI: susceptibility weighted imaging

**Figure 5:**
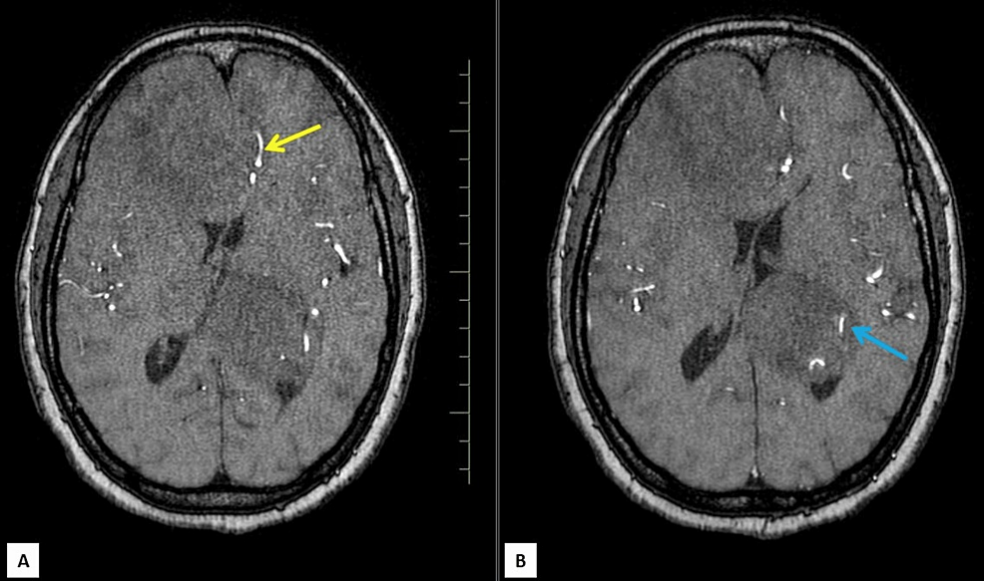
Magnetic resonance angiogram time-of-flight (TOF) images (A,B) show displacement of both the anterior cerebral arteries to the left (yellow arrow) and small blood vessels seen traversing the intraventricular lesion (blue arrow)

The dural-based lesion was fairly defined showing T1-isointense and T2/FLAIR heterogeneously hypointense signals. It was causing mass effect in the form of compression of the adjacent brain parenchyma and frontal horns of bilateral lateral ventricles with effacement of sulcal spaces and mild cortical buckling. Subfalcine herniation to the left was seen. Surrounding significant perilesional T2/FLAIR white matter hyperintensities were seen signifying edema (Figures 2,3). There was no evidence of internal susceptibility weighted imaging (SWI) hypointensities (Figure 4). The lesion was seen displacing both the anterior cerebral arteries to the left, with a few small blood vessels seen traversing the mass and feeder arising likely from branches of the right anterior cerebral artery (Figure 5).

**Figure 6:**
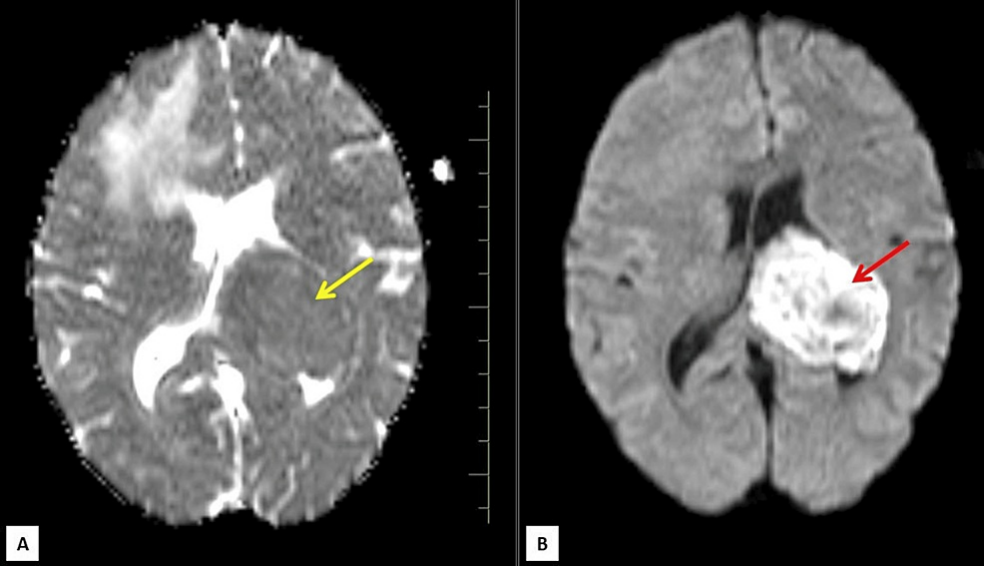
Axial ADC (A) and DWI (B) MRIs show high signal intensity (red arrow) with no obvious ADC hypointensity (yellow arrow) in the intraventricular lesion ADC: apparent diffusion coefficient; DWI: diffusion-weighted imaging

The intraventricular lesion appeared T1 isointense to gray matter and T2/FLAIR heterogeneously hyperintense (Figures 1,2). Diffusion-weighted images along with apparent diffusion coefficient (ADC) maps revealed high DWI signal intensity with no obvious ADC hypointensity (Figure 6). The lesion was seen causing compression of the adjacent brain parenchyma and effacement of adjacent sulcal spaces, leading to a mild asymmetrical widening of the frontal horn and atria of the left lateral ventricle. It was seen extending superiorly to the adjacent periventricular region abutting the left lateral and inferior aspect of corpus callosum (e.g., body, isthmus, and splenium) and, inferomedially, was seen abutting the left thalamus. There was no perilesional edema or internal calcifications (Figures 2-4). Multiple small blood vessels were seen traversing the mass, with the feeder likely arising from the branches of the left posterior cerebral artery (Figure 5).

**Figure 7:**
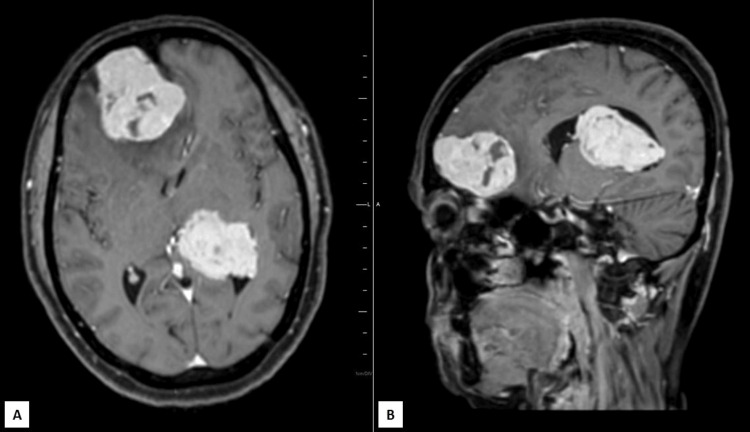
Postcontrast T1W axial (A) and sagittal (B) MRIs show intense homogenous enhancement with internal necrotic areas in both dural and intraventricular lesions

Magnetic resonance (MR) spectroscopy revealed high levels of alanine and postcontrast T1 images of both lesions revealed intense homogenous enhancement, with internal necrotic areas (Figure 7). The aforementioned findings led to the diagnosis of coexisting meningiomas located in the dura of the frontal region and left ventricle.

**Figure 8:**
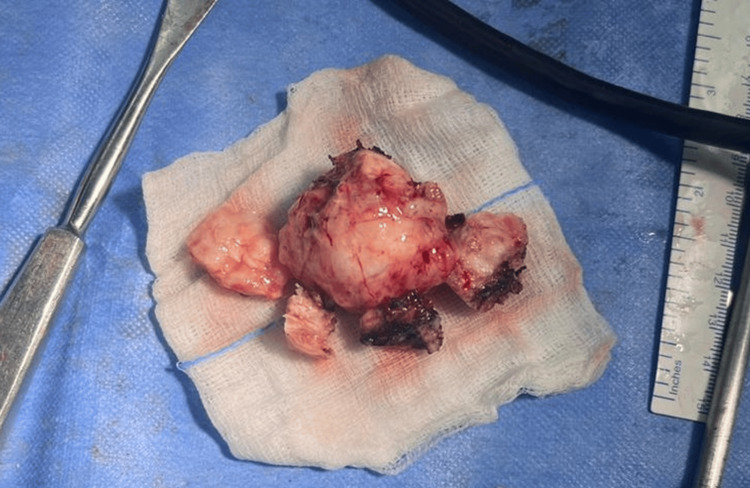
Gross specimen of the dural-based meningioma

**Figure 9:**
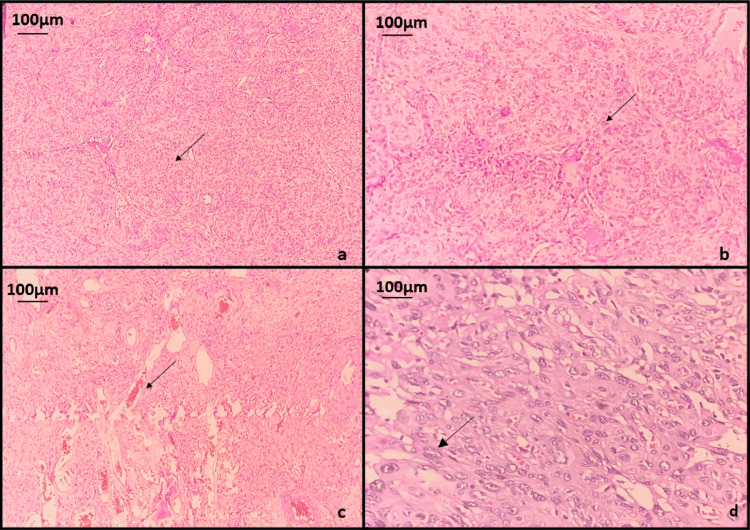
Photomicrographs of H&E-stained sections showing (a) neoplasm composed of cells arranged in sheets and whorls (black arrow) (40x); (b) meningothelial cells with moderate eosinophilic cytoplasm, indistinct cytoplasmic borders and uniform round to slightly oval nuclei (black arrow) (100x); (c) blood vessels of varying sizes intervening the cells (black arrow) (100x); (d) cells with mild to moderate nuclear pleomorphism (black arrow) (400x)

This diagnosis was later confirmed through histopathology after surgically removing both tumors (Figure 8). A histopathological analysis of the tissue specimen obtained during surgical resection confirmed the diagnosis of meningothelial meningioma (WHO grade 1). Histopathological sections of the dural lesion showed multiple fragments of tissue showing a neoplasm comprising sheets and whorls of meningothelial cells with moderate eosinophilic cytoplasm, indistinct cytoplasmic borders, and uniform round to slightly oval nuclei with many blood vessels of varying sizes intervening the cells. Focal areas show cells with mild to moderate nuclear pleomorphism. No mitotic figures or necrosis were noted (Figure 9).

Both tumors were successfully removed with a gross total resection, and there were no postoperative complications. The patient was sent to a more advanced medical facility for additional treatment, which included chemotherapy and radiation. However, the patient was lost to follow-up.

## Discussion

Meningiomas that develop from the meninges, the protective linings that surround the brain and spinal cord, are often slow-growing tumors and originate from arachnoid cap cells, which are found naturally close to dural margins and venous sinuses [4]. They are found in the dura mater, which is the outermost layer of the meninges, and intraventricular meningiomas account for only a small percentage of all meningiomas. According to reports, only 1%-10% of patients have multiple meningiomas (MM), while more recent data point to a higher occurrence and constitute a separate clinical entity with distinct etiologies, such as radiation-induced, familial, and sporadic.

Although the pathophysiology of multiple meningiomas is unknown, suggestions include independent development in different sites through distinct genetic events and the "monoclonal hypothesis," wherein many distinct meningiomas are precipitated by a modified neoplastic clone with subarachnoid seeding [5].

Only 0.5-3% of meningiomas occur in an intraventricular location. The lateral ventricle accounts for 88.4% of cases, whereas the third and fourth ventricles account for 2.9% and 8.7% of cases, respectively. They are typically seen inside the atrium [6]. The source of intraventricular meningiomas is arachnoid cells seated in the choroid plexus. The mesenchymal stroma of the choroid plexus, which has meningothelial inclusion bodies in the tela choroidea, is the source of lateral ventricle meningiomas. Meningiomas of the fourth ventricle originate from the tela choroidea, while tumors of the third ventricle develop from the tela of the velum interpositum [7]. Therefore, the simultaneous presence of these two distinct tumor types in a single patient is an unusual finding.

The most commonly acknowledged mechanism for this uncommon coexistence is the dissemination of tumor cells through cerebrospinal fluid or the bloodstream [8]. The notable female bias observed in sporadic meningiomas could be explained by the presence of progesterone receptors found in up to 72% to 90% of meningiomas. Because of increased blood progesterone levels during pregnancy, female patients may exhibit increasing symptoms [9].

Although some meningiomas are linked to genetic disorders or mutations, most are sporadic. Even sporadic meningiomas are usually caused by a mutation in chromosome 22 (also known as the INI1, sis oncogene, or Merlin tumor suppressor gene). Neurofibromatosis type 2, commonly known as multiple inherited schwannomas or MISME syndrome, is caused by the same mutation. In 89% of intraventricular meningiomas, chromosome 22q is lost, and in 44%, chromosome 1p is lost [10]. The most common genetic modification identified in intraventricular meningiomas is an NF2 mutation [11].

The WHO CNS5 grading system for meningiomas aligns closely with the WHO 2016 system, incorporating three malignancy grades (CNS WHO grades 1-3) determined by histopathology or subtype. Meningiomas are now considered a unified tumor type with 15 subtypes, and the grading approach has shifted to within-tumor grading, irrespective of subtype. Notably, choroid and clear-cell meningiomas, identified as having a heightened recurrence risk, are classified as grade 2. Brain-invasive meningiomas, associated with an elevated recurrence risk, are now categorized as atypical meningiomas (CNS WHO grade 2), aligning with the WHO 2016 classification. Rhabdoid and papillary meningiomas, although displaying aggressive behavior, are no longer automatically designated as grade 3. Instead, they are classified as meningiomas in general [12].

Meningiomas can be divided into NF2 (neurofibromatosis type 2) and non-NF2-mutated categories. NF2-mutated convexity meningiomas, predominantly exhibiting fibroblastic and transitional phenotypes, are more frequently classified as CNS WHO grade 2 and 3. Conversely, non-NF2 meningiomas, often located in the skull, present meningothelial and secretory phenotypes. Aggressive atypical meningiomas and those with borderline grades 2-3 histopathology warrant genetic analysis, with TERTp mutation and homozygous CDKN2A/B loss indicating grade 3 tumors. H3K27me3 loss signals more aggressive behavior. DNA methylation is effective at stratifying meningiomas into classes that more precisely identify patients at a high risk of recurrence than histopathology alone. The future diagnostic approach to meningiomas is anticipated to involve molecular classification based on copy number variation, point mutations, methylation, and transcriptomic and proteomic data. Histopathological grading remains a significant prognostic factor in human meningiomas, crucial for informing therapeutic strategies and follow-up protocols [12].

In this case, the patient presented with chronic headaches, which prompted further investigation with a brain MRI. The imaging revealed two supratentorial lesions, one located in the dura of the frontal region and the other within the left lateral ventricle. The dural lesion exhibited characteristics typically associated with meningiomas, including a broad-based attachment, mass effect on adjacent structures, and perilesional edema. On the other hand, the intraventricular lesion, showed distinct features within the ventricular space, causing compression of surrounding brain tissue.

Histopathological examination confirmed the diagnosis of coexisting meningiomas in both locations. Atypical meningiomas are a subtype of meningiomas that exhibit more aggressive behavior compared to the more common benign variants. The presence of internal necrotic areas and the intense homogeneous enhancement observed in the post-contrast MRI images further supported the atypical nature of the tumors. MR spectroscopy revealed elevated levels of alanine, which is a metabolic marker associated with tumor activity.

In general, it has been discovered that meningiomas have higher levels of alanine than the normal brain and other tumors, and it is currently being utilized to differentiate meningiomas from gliomas and metastases. It is uncertain why meningiomas show a strong alanine peak; however, it is found that extracts of dural tissue contain large amounts of this metabolite. Meningiomas can create as much as twice as much as other tumors when it comes to the production of alanine, or it can be a by-product that accumulates in the tissue [13].

The lack of weakness in our patient, despite the presence of large intracranial space-occupying lesions (ICSOLs), may be attributed to the location of the meningioma. Meningiomas, especially those situated in extra-axial locations such as the dura mater or ventricular system, may exert pressure effect primarily on adjacent structures without directly infiltrating or compressing critical neurological pathways, unlike infiltrative lesions such as high-grade gliomas, which infiltrate and disrupt neural tissue. Secondly, the chronic and insidious nature of the tumors' growth may have enabled compensatory mechanisms such as neuroplasticity to lessen their negative effects on brain function.

The treatment of choice for meningiomas is surgical resection, and, in this case, both tumors were successfully removed [14]. However, because of the atypical nature of the meningiomas and the potential for residual tumor cells, the patient was referred to a higher medical center for further management, and we were unable to follow up with the patient. Radiotherapy and chemotherapy may be recommended as adjuvant treatments to reduce the risk of recurrence or progression of the disease.

Gross complete resection is advised for big, asymptomatic lesions larger than 3 cm in young, healthy surgical candidates as it is typically curative. Because vascular control is often deep within the tumor, total resection may not always be possible. In these cases, preoperative endovascular embolization should be tried. Partial resection followed by adjuvant radiosurgery is a safe approach if vascular control is difficult to obtain. In cases when the diagnosis is ambiguous, poor surgical candidates get a needle biopsy, which is followed by radiosurgery and when the diagnosis is obvious, radiosurgery is adequate on its own. The size, laterality, and ventricle wherein the tumor is placed determine the surgical strategy. Surgery techniques include the posterior interhemispheric transcingular (via dissection of precuneus or cingulate gyrus), transcallosal interhemispheric, intraparietal sulcus, and transtemporal approaches at the posterior part of the middle temporal gyrus [7].

The uniqueness of this case lies in the rare coexistence of dural and intraventricular meningiomas within the same patient, and the absence of significant neurological deficits despite the presence of large ICSOLs further distinguishes this case, underscoring the need for further research into the neurophysiological mechanisms underlying the interaction between tumor growth and neurological function.

## Conclusions

The coexistence of dural and intraventricular meningiomas is an extremely rare finding, with possible mechanisms for this manifestation being dissemination through cerebrospinal fluid or bloodstream. This case highlights the importance of comprehensive imaging techniques, such as MRI, in the evaluation and diagnosis of complex brain tumors. The distinct radiological characteristics and histopathological confirmation allowed for accurate identification of meningiomas in both the dural and intraventricular locations. Surgical resection is the treatment of choice for meningiomas, with complete resection recommended for large, asymptomatic lesions. Surgical techniques vary based on tumor size, location, and accessibility. Adjuvant treatments such as radiotherapy and chemotherapy may be considered to reduce recurrence or disease progression. Prognosis depends on factors such as extent of resection, molecular characteristics, and histopathological grade.
